# Local and Systemic Overexpression of COMP-Ang1 Induces Ang1/Tie2-Related Thrombocytopenia and SDF-1/CXCR4-Dependent Anemia

**DOI:** 10.1093/stmcls/sxac080

**Published:** 2022-11-11

**Authors:** Hyun-Jaung Sim, Govinda Bhattarai, Min-Hye Kim, Han-Sol So, Sher Bahadur Poudel, Eui-Sic Cho, Sung-Ho Kook, Jeong-Chae Lee

**Affiliations:** Cluster for Craniofacial Development and Regeneration Research, Institute of Oral Biosciences and School of Dentistry, Jeonbuk National University, Jeonju, South Korea; Department of Bioactive Material Sciences, Research Center of Bioactive Materials, Jeonbuk National University, Jeonju, South Korea; Cluster for Craniofacial Development and Regeneration Research, Institute of Oral Biosciences and School of Dentistry, Jeonbuk National University, Jeonju, South Korea; Department of Bioactive Material Sciences, Research Center of Bioactive Materials, Jeonbuk National University, Jeonju, South Korea; Department of Bioactive Material Sciences, Research Center of Bioactive Materials, Jeonbuk National University, Jeonju, South Korea; Department of Molecular Pathobiology, New York University College of Dentistry, New York, NY, USA; Cluster for Craniofacial Development and Regeneration Research, Institute of Oral Biosciences and School of Dentistry, Jeonbuk National University, Jeonju, South Korea; Cluster for Craniofacial Development and Regeneration Research, Institute of Oral Biosciences and School of Dentistry, Jeonbuk National University, Jeonju, South Korea; Department of Bioactive Material Sciences, Research Center of Bioactive Materials, Jeonbuk National University, Jeonju, South Korea; Cluster for Craniofacial Development and Regeneration Research, Institute of Oral Biosciences and School of Dentistry, Jeonbuk National University, Jeonju, South Korea; Department of Bioactive Material Sciences, Research Center of Bioactive Materials, Jeonbuk National University, Jeonju, South Korea

**Keywords:** excessive angiogenesis, megakaryopoiesis, erythrocyte maturation, GATA-1, SDF-1, Ang1/Tie2 signaling axis

## Abstract

While supplemental angiopoietin-1 (Ang1) improves hematopoiesis, excessive Ang1 induces bone marrow (BM) impairment, hematopoietic stem cell (HSC) senescence, and erythropoietic defect. Here, we examined how excessive Ang1 disturbs hematopoiesis and explored whether hematopoietic defects were related to its level using *K14-Cre;c-Ang1* and *Col2.3-Cre;c-Ang1* transgenic mice that systemically and locally overexpress cartilage oligomeric matrix protein-Ang1, respectively. We also investigated the impacts of Tie2 inhibitor and AMD3100 on hematopoietic development. Transgenic mice exhibited excessive angiogenic phenotypes, but *K14-Cre;c-Ang1* mice showed more severe defects in growth and life span with higher presence of Ang1 compared with *Col2.3-Cre;c-Ang1* mice. Dissimilar to *K14-Cre;c-Ang1* mice, *Col2.3-Cre;c-Ang1* mice did not show impaired BM retention or senescence of HSCs, erythropoietic defect, or disruption of the stromal cell-derived factor 1 (SDF-1)/CXCR4 axis. However, these mice exhibited a defect in platelet production depending on the expression of Tie2 and globin transcription factor 1 (GATA-1), but not GATA-2, in megakaryocyte progenitor (MP) cells. Treatment with Tie2 inhibitor recovered GATA-1 expression in MP cells and platelet production without changes in circulating RBC in transgenic mice. Consecutive AMD3100 administration not only induced irrecoverable senescence of HSCs but also suppressed formation of RBC, but not platelets, via correlated decreases in number of erythroblasts and their GATA-1 expression in B6 mice. Our results indicate that genetic overexpression of Ang1 impairs hematopoietic development depending on its level, in which megakaryopoiesis is preferentially impaired via activation of Ang1/Tie2 signaling, whereas erythropoietic defect is orchestrated by HSC senescence, inflammation, and disruption of the SDF-1/CXCR4 axis.

## Graphical Abstract

**Graphical Abstract:**
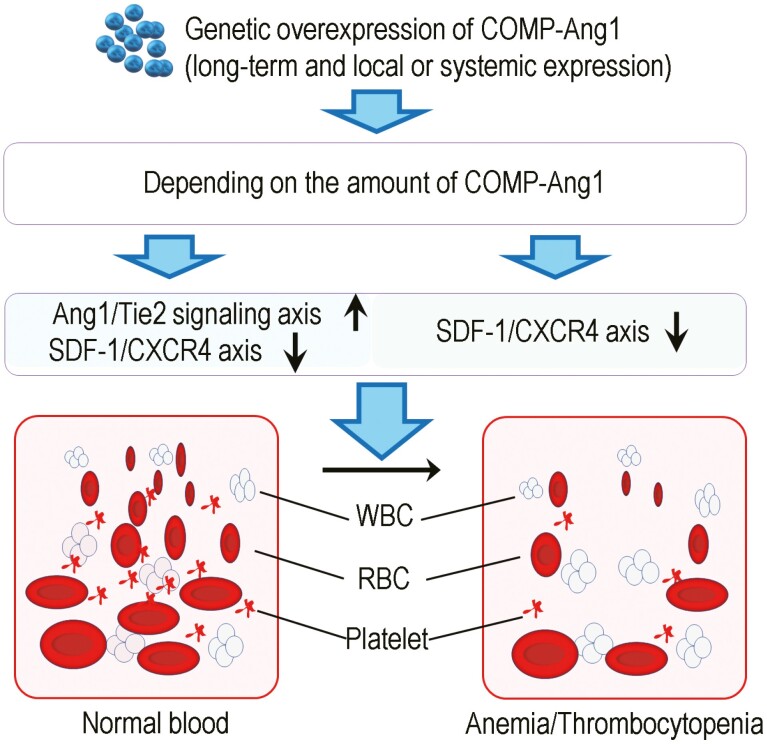

Genetic overexpression of COMP-Ang1 impairs BM microenvironment, induces HSC senescence, and leads to anemic and thrombocytopenic disorders depending on its systemic and local overexpression through a dysregulated activation of the Ang1/Tie2 signaling and/or SDF-1/CXCR4 retention axis.

**Significant Statement**

The underlying mechanisms through which transgenic overexpression of COMP-Ang1 regulates megakaryopoietic and erythropoietic developments were investigated. This study demonstrates that genetic overexpression of COMP-Ang1 preferentially impairs megakaryopoiesis via activation of Ang1/Tie2 signaling. The current findings also highlight that erythropoietic defect by excessive COMP-Ang1 is orchestrated by HSC senescence, inflammation, and disruption of the SDF-1/CXCR4 axis.

## Introduction

Angiopoietin-1 (Ang1) controls vascular development and maintenance through its interaction with its specific receptor, Tie2.^1^ Although the exact role of Ang1 on hematopoiesis is not yet well understood, numerous studies have indicated that the Ang1/Tie2 signaling axis plays essential roles in the long-term adhesion of hematopoietic stem cells (HSCs) to bone marrow (BM) niches, maintenance of HSCs in the BM, and hematopoietic development.^2-6^ Studies have also demonstrated the crucial roles of the Ang1/Tie2 signaling axis in regulating proliferation, differentiation, and maturation of megakaryocytes^7,8^ and erythroblasts.^9,10^ These studies suggest a relationship between angiogenesis and hematopoietic development, in which the Ang1/Tie2 axis acts as a key signaling regulator.

Total body irradiation, chemotherapy, and/or conditioned BM transplantation often impair the BM microenvironments that are critical for BM retention and functional maintenance of HSCs and hematopoietic progenitor cells (HPCs).^11^ While supplemental Ang1 shows various impacts on endothelial cell survival, vascular anti-inflammation, and neovascularization,^12-14^ studies have also supported its beneficial role in hematopoiesis. Administration of Ang1 via plasmid injection facilitated hematopoietic recovery in radiated mice by increasing Tie2 and Notch expression.^15^ Genetic overexpression of Ang1 in type I collagen (*Col1a1*)-expressing osteoblasts increased long bone mass and mineral density.^16^ These reports not only indicate an interaction among angiogenesis, hematopoiesis, and osteogenesis but also imply the induction of BM microenvironmental rebuilding and hematopoietic recovery in response to the supplementation with Ang1.

Cartilage oligomeric matrix protein (COMP)-Ang1 is a chimera of Ang1 that has higher solubility and stability with greater angiogenic potential than native Ang1.^17^ Long-term administration of COMP-Ang1 by adenoviral vector expression exerted greater therapeutic benefits than supplementation with the recombinant human (*rh*) protein.^18^ We previously generated transgenic mice that overexpress COMP-Ang1 in Keratin 14 (*K14*)-expressing cells and investigated whether genetic supplementation with COMP-Ang1 also positively affected bone growth and function of HSCs. The transgenic mice exhibited severe and excessive angiogenesis in the body together with an impaired BM microenvironment and HSC senescence.^19^ The mice also exhibited a defect in erythroblastic maturation, increased production of inflammatory cytokines, dysregulated expression of globin transcription factor 1 (GATA-1), and imbalanced retention of the stromal cell-derived factor-1 (SDF-1)/CXCR4 axis in the BM.^20^ Our previous findings indicated that persistent and prolonged supplementation with Ang1 by transgenic overexpression did not induce a benefit on the BM microenvironment and hematopoiesis, rather than it dysregulates hematopoietic development via an imbalanced activation or disruption of the Ang1/Tie2 signaling axis, SDF-1/CXCR4 retention axis, GATA-1 expression, or all of these mechanisms.

Here, we investigated the underlying mechanisms through which transgenic overexpression of COMP-Ang1 regulates megakaryopoietic and erythropoietic developments. We explored whether erythropoiesis and megakaryopoiesis are differently affected in relation to the amount of COMP-Ang1 generated. We generated mice that express local COMP-Ang1 in *Col1a1*-expressing cells and compared the results with hematopoietic events derived from mice that express Ang1 in *K14*-expressing cells (Supplementary Fig. S1). To clarify the molecular mechanisms by which the Ang1/Tie2 signaling axis, the SDF-1/CXCR4 retention axis, or both affect hematopoietic development, we determined the impacts of Tie2 inhibitor, *rh*COMP-Ang1, or AMD3100 treatment in transgenic mice, B6 mice, and/or irradiated recipients. Our results demonstrated that transgenic overexpression of COMP-Ang1 impairs GATA-1-related megakaryopoiesis and platelet production via activation of the Ang1/Tie2 signaling axis regardless of the amount of Ang1. Our findings also provide evidence that systemic, but not local, overexpression of COMP-Ang1 results in defects in erythrocyte maturation and red blood cell (RBC) production, and this defect is closely linked to HSC senescence and disruption of the SDF-1/CXCR4 retention axis.

## Materials and Methods

### Study Approval and Animals

This study was carried out in strict accordance with the recommendations in the Guide for Animal Care and Use of Jeonbuk National University. Before experiments, all procedures were approved by the University Committee on Ethics in the Care and Use of Laboratory Animals according to the ARRIVE guidelines. We generated *K14* (*2.2 kb*)*-Cre;inducible-COMP-Ang1-transgenic* (*K14-Cre;c-Ang1*) and *Col1a1* (*2.3 kb*)*-Cre;inducible-COMP-Ang1-transgenic* (*Col2.3-Cre;c-Ang1*) mice by crossing the *K14-Cre*^21,22^ and the *Col2.3-Cre* mice with *inducible-COMP-Ang1-transgenic* mice,^23^ respectively (Supplementary Fig. S1). Here, we used the *Col2.3-Cre* mice, but not *Col3.6-Cre* mice, in generating COMP-Ang1 transgenic mice. This was because the mice crosslinked with *Col2.3-Cre* mice expressed COMP-Ang1 in a relatively local and osteoblast-specific manner than did the mice with *Col3.6-Cre* mice. COMP-Ang1 transgenic mice were backcrossed with C57BL6 strain for more than 3 generations. Mouse offspring were genotyped by polymerase chain reaction (PCR) analysis following the methods described previously^21,22^ and used in all experiments regardless of gender. All animals were cared for in the guidelines of the Animal Care Committee of Jeonbuk National University.

### Treatments with Tie2 Kinase Inhibitor, *rh*COMP-Ang1, or AMD3100

Megakaryocytic progenitor (MP) cells were isolated from C57BL/6 (B6) mice (3-weeks-old, Orient Bio, Daejeon, South Korea) and incubated with various concentrations (0–1000 ng/mL) of *rh*COMP-Ang1 or in combination with Tie2 kinase inhibitor (250 nM). After incubation, these cells were processed for flow cytometric determination of GATA-1 expression in MP cells. Parts of transgenic mice and their littermate controls were peritoneally injected with Tie2 kinase inhibitor (10 mg/kg), and levels of circulating platelets and GATA-1 expression in BM-conserved MP cells were determined. Alternatively, B6 mice (4-weeks-old) received subcutaneous injection of AMD3100 (0-5 mg/kg/day; Sigma-Aldrich Co. LLC) diluted in sterile endotoxin-free phosphate buffered saline (PBS) for 1 day or 21 consecutive days. Cells from the BM, spleen, and peripheral blood (PB) were collected 12 h after the last administration of AMD3100, and the role of SDF-1/CXCR4 axis on hematopoietic development was further investigated.

### Cell Preparation and Flow Cytometry

Cells were isolated from BM, spleen, and PB of transgenic mice, littermate controls, or B6 mice. After removal of RBC and washing with PBS, frequencies of cells were analyzed using a flow cytometer (BD Calibur or BD Aria, BD Bioscience) installed at the Center for University-Wide Research Facilities of Jeonbuk National University. Cell populations were sequentially gated using FlowJo software program (FLOWJO; Ashland, OR). In brief, populations of Lin^-^Sca-1^+^c-Kit^+^ (LSK) cells and CD150^+^CD48^-^LSK cells were phenotypically identified using following antibodies: lineage markers PE-Cy7-conjuaged anti-CD3 (cat.#552774), anti-B220 (CD45R; cat.#552772), anti-CD11b (cat.#552850), anti-Gr-1 (cat.#552958), or anti-TER-119 (cat.#557853) (All of these markers were from BD Biosciences); FITC-conjugated anti-Sca-1 (cat.#557405; BD Biosciences) or PE-conjugated anti-Sca-1 (cat.#553108; BD Biosciences); APC-conjugated anti-c-Kit (cat.#553356; BD Biosciences); perCP/Cy5.5-conjuated anti-CD150 (cat.#46-1502; eBiosciences); and APC-Cy7conjugated anti-CD48 (cat.#561826; BD Biosciences). Senescence-associated-β-galactosidase (SA-β-gal) activity in LSK, CD150^+^CD48^-^LSK, or Lin^-^Sca-1^+^CD29^+^CD105^+^ cells that had been already incubated with the cell surface markers were analyzed using C_12_FDG (cat.#I2904; Molecular Probes). In addition, HPCs including granulocyte-monocyte progenitors (GMPs), common myeloid progenitors (CMPs), and megakaryocyte-erythroid progenitor (MEPs) were defined using PE-conjugated anti-FcR (BD Biosciences), perCP/Cy5.5-conjuated anti-CD34 (BioLegend), or PE-conjugated anti-IL-7R (BD Biosciences) as a basis for LSK cell markers. Erythroblasts at four stages (ProE, proerythroblasts; BasoE, basophilic erythroblasts; PolyE, polychromatophilic; OrthE, orthochromatophilic erythroblasts) were discriminatively gated with PE- or FITC-conjugated anti-CD71 (BD Biosciences) and PE-Cy7-conjugated anti-TER-119 (BD Biosciences) as described previously.^20^ MP cells were analyzed with PE-Cy7-conjugated anti-lineage markers (BD Biosciences), APC-Cy7-conjugated anti-Sca-1 (cat.#561681; BD Biosciences), Texas Red-conjugated anti-c-Kit (cat.#562417; BD Biosciences), PE-conjugated anti-FcR (cat.#553141; BD Biosciences), APC-conjugated anti-CD9 (cat.#17-0091-82; eBioscience), and PerCP-eFluor-conjugated anti-CD41 (cat.#46-0411-82; eBioscience). In this study, MP cells were defined as Lineage^−^, IL7Rα^−^, Sca-1^−^, c-Kit^+^, CD9^+^, FcR^low^, and CD41^+^ cells. Levels of GATA-1 (cat.#939101), GATA-2 (cat.#614003), and CXCR4 (cat.#85578) in BM- and spleen-conserved MP cells were determined with PE-conjugated antibodies (Cell Signaling or BioLegend) after fixation and permeabilization. Numbers of Ki-67-positive BM-derived MP cells were measured using FITC-conjugated anti-Ki-67 antibody (cat.#556026; BD Pharmingen^TM^). BM-derived hematopoietic stem progenitor cells (HSPCs) expressing Tie2 were measured using flow cytometer (BD Aria; BD Biosciences) after staining with PE-conjugated antibody (cat.#7403; Cell Signaling). To evaluate the impacts of COMP-Ang1 overexpression in *Col2.3-Cre;c-Ang1* mice on BM-mesenchymal stem cells (MSCs), populations of Lin^-^Sca-1^+^CD29^+^CD105^+^ cells were phenotypically identified as MSCs using the same PE-Cy7-conjugated lineages as used for identification of hematopoietic cells. Unless specified otherwise, experiments using transgenic mice and their littermate controls were performed at 3 weeks of age.

### Assays to Evaluate the Effect of AMD3100 Treatment

BM, spleen, and PB cells were collected from B6 mice administered with AMD3100 (0–5 mg/kg) for 1 day or consecutive 21 days. The frequencies of BM- and PB-conserved cells were analyzed using a flow cytometer (BD Calibur or BD Aria, BD Bioscience). The frequencies of erythroblasts at 4 stages and MP cells and GATA-1 expression in these cells were determined as described above. BM cells (4 × 10^5^ cells) were also collected from PBS (vehicle)- or AMD3100 (5 mg/kg)-injected mice (CD45.2). These cells were transplanted by tail vein injection together with BM cells (4 × 10^5^ cells) of competitor mice (CD45.1) into recipients (CD45.1/2) that were lethally irradiated with 10 Gy as described above. Donor cell-derived repopulating capacity was assessed using PB of the recipients after 5 months of transplantation. In addition, levels of p15, p16, p19, and p21 mRNAs in BM cells of vehicle- and consecutive AMD3100 (5 mg/kg)-treated mice were determined by real time PCR analysis. In this assay, cDNA was synthesized using 1 µg of BM cell-derived total RNA for each sample and AmpiGene^TM^ cDNA Synthesis kit (Enzo Life Sciences). Power SYBR^®^ Green PCR Master Mix (Life Technologies) was used to detect accumulated PCR products during cycling using ABI StepOnePlus sequence detection system (Applied Biosystems). Oligonucleotide primers specific to *p15*, *p16*, *p19*, *p21*, and *GAPDH* were designed as follows; ccctgccacccttaccaga (forward) and cagatacctcgcaatgtcacg (reverse) for *p15*, gtcgcaggttcttggtcact (forward) and tctgcaccgtagttgagcag (reverse) for *p16*, gccggcaaatgatcatagag (forward) and cagcaagagctggatcagaa (reverse) for *p19*, tgtccgtcagaacccatc (forward) and aaagtcgaagttccatcgcc (reverse) for *p21*, and gacggccgcatcttcttgt (forward) and cacaccgaccttcaccatttt (reverse) for *GAPDH*.

### Other Methods

For details of other methods including blood vessel measurement, transplantation assay, Annexin V/propidium iodide (PI) staining, circulating blood cell counting, immunohistochemistry (IHC), and enzyme-linked immunosorbent assay (ELISA) see the Supporting Information.

### Statistical Analyses

All data are expressed as the mean ± SD and analyzed using SPSS program (ver. 12.0). Differences between two groups were analyzed by unpaired Student’s *t*-test (*n* ≥ 6) or by a non-parametric test (Wilcoxon *t*-test, *n* < 6). One-way ANOVA followed by the Scheffe’s multiple range test was used for multiple comparisons among more than two groups. The Kolmogorov–Smirnov test was used to test the normality of data sets. A value of *P* < 0.05 was considered statistically significant.

## Results

### Transgenic Systemic and Local Overexpression of COMP-Ang1 Affects BM Retention and Senescence of HSCs, BM Levels of Inflammatory Cytokines and SDF-1, RBC Production, and Survival

**Figure 1.**
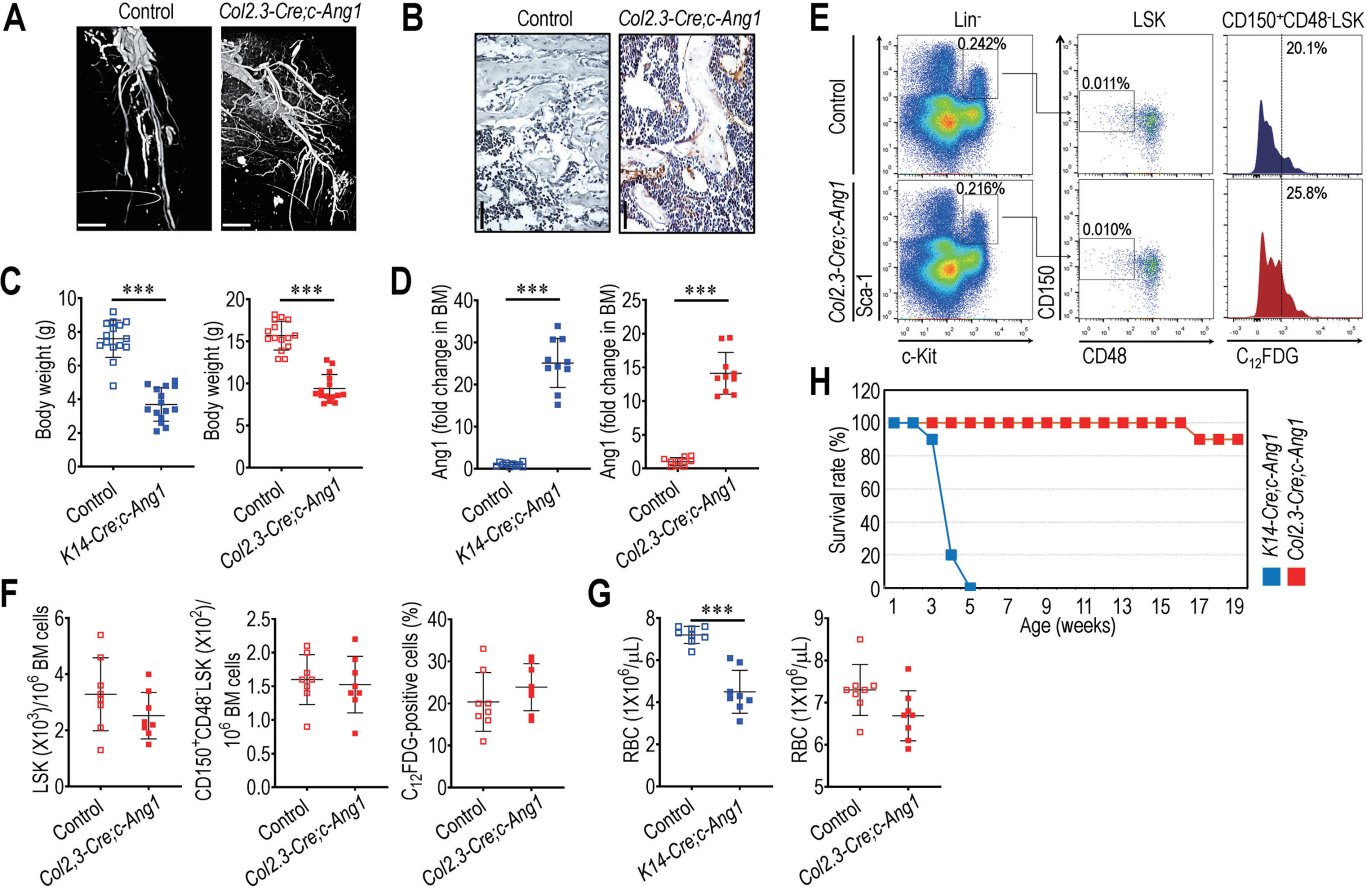
Genetic overexpression of COMP-Ang1 affects cellular and biological phenotypes based on the expression systems involved. **(A)** The µCT images of blood vessels around the right femur of *Col2.3-Cre;c-Ang1* mice and littermate controls after Microfil injection and decalcification (Scale bar, 200 μm). (**B)** Immunohistochemical assay showing CD31 expression in BM of *Col2.3-Cre;c-Ang1* mice and littermate controls (Scale bar, 200 μm). (**C)** Body weight (*n* = 15) and (**D**) Ang1 level in BM supernatants (*n* = 10) of transgenic mice and littermate controls at 3 weeks of age. (**E)** A representative flow cytometric result and (**F**) mean numbers of LSK and CD150^+^CD48^-^LSK cells along with C_12_FDG-positive CD150^+^CD48^-^LSK cells (%) in the BM of *Col2.3-Cre;c-Ang1* mice and littermate controls (*n* = 8). (**G)** Circulating RBC level in transgenic mice and their littermate controls at 3 weeks of age (*n* = 8). (**H)** Survival rates (%) of the *K14-Cre;c-Ang1* and *Col2.3-Cre;c-Ang1* mice at the indicated age (*n* = 10). Data represent mean ± S.D. ^***^P < 0.001 vs. the controls by unpaired Student’s *t*-test.

Similar to the *K14-Cre;c-Ang1* mice,^19,20^ the *Col2.3-Cre;c-Ang1* mice exhibited more branched and enlarged vessels around the hind limbs (Fig. 1A) and a greater number of CD31-positive cells in the BM (Fig. 1B) compared with their littermate controls. Both the *K14-Cre;c-Ang1* and *Col2.3-Cre;c-Ang1* mice at 3 weeks of age showed a lower body weight (Fig. 1C) and greater BM level of Ang1 (Fig. 1D) compared with their littermate controls. However, the numbers of BM LSK, CD150^+^CD48^-^LSK, and C_12_FDG (a β-gal substrate)-positive CD150^+^CD48^-^LSK cells in *Col2.3-Cre;c-Ang1* mice were comparable to those in littermate controls (Fig. 1E, 1F). The frequencies of LSK and CD150+CD48-LSK cells in PB of *Col2.3-Cre;c-Ang1* mice were also similar to those of littermate controls (Supplementary Fig. S2). The *Col2.3-Cre;c-Ang1* mice did not exhibit any alterations in the numbers of BM MSCs (Supplementary Fig. S3A) and C_12_FDG-positive MSCs (Supplementary Fig. S3B), and the ability of MSCs from these mice to form colonies were unchanged compared with their littermate controls (Supplementary Fig. S3C). Compared with littermate controls, *Col2.3-Cre;c-Ang1* mice did not show any changes in the levels of inflammatory cytokines (IL-1α, IL-6, IFN-γ, and TNF-α) and SDF-1 in BM supernatants (Supplementary Fig. S4A, S4B). In addition, the total numbers of BM erythroblasts and GATA-1-expressing erythroblasts (Supplementary Fig. S4C, S4D) and redder color in femurs and tibias (Supplementary Fig. S4E) in *Col2.3-Cre;c-Ang1* mice were not different from those in their littermate controls. While *K14-Cre;c-Ang1* mice contained a significantly lower level of circulating RBC compared with their littermate controls (*P* < 0.001), the *Col2.3-Cre;c-Ang1* mice showed a similar number of RBC to that in littermate controls (Fig. 1G). Furthermore, *K14-Cre;c-Ang1* mice, but not *Col2.3-Cre;c-Ang1* mice, showed severe mortality at a young stage (Fig. 1H). These results together with our previous reports^19,20^ indicate that transgenic overexpression of COMP-Ang1 increases angiogenesis-associated phenotypes regardless of its local and systemic expression, whereas BM retention and senescence of HSCs, hematopoietic development, BM levels of inflammatory cytokines and SDF-1, and survival rate were affected in relation to the amount of COMP-Ang1.

### Genetic Overexpression of COMP-Ang1 Decreases BM Frequency of MP Cells and Circulating Platelet Levels, but does not affect Proliferation Capacity of MP Cells

**Figure 2.**
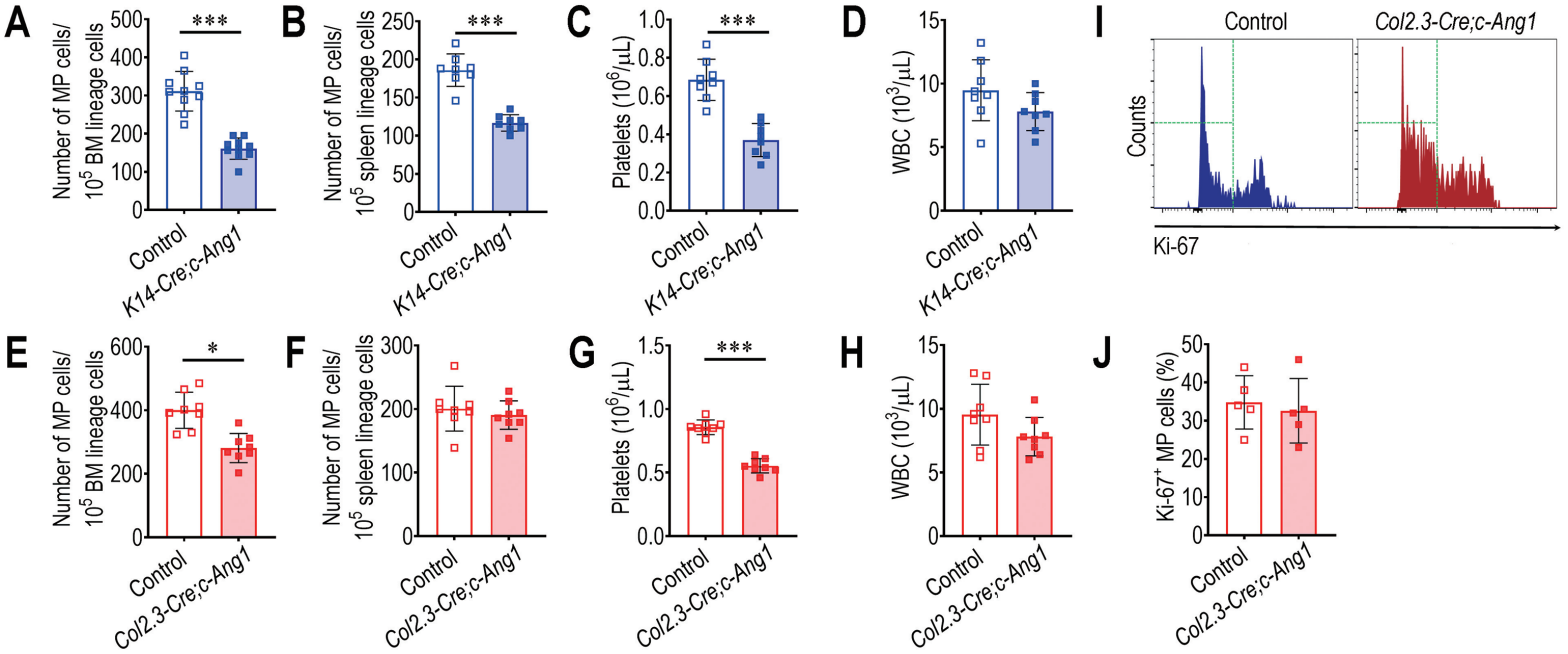
Excessive COMP-Ang1 decreases number of MP cells conserved in BM and/or spleen and diminishes circulating platelet level. Numbers of MP cells conserved in (**A**) BM (*n* = 10) and (**B**) spleen (*n* = 8) of *K14-Cre;c-Ang1* mice and littermate controls. Numbers of (**C**) circulating platelets and (**D**) WBC in *K14-Cre;c-Ang1* mice and littermate controls (*n* = 8). Numbers of (**E**) BM- or (**F**) spleen-conserved MP cells, (**G**) platelets, and (**H**) WBC in *Col2.3-Cre;c-Ang1* mice and littermate controls (*n* = 8). (**I)** Flow cytometric histogram representing Ki-67-positive MP cells in the BM of *Col2.3-Cre;c-Ang1* mice and littermate controls, and (**J**) the mean number of Ki-67-positive MP cells (%) (*n* = 5). Data represent mean ± S.D. ^*^P < 0.05; ^**^P < 0.01; ^***^P < 0.001 vs the controls by unpaired Student’s *t*-test.

We next explored how transgenic COMP-Ang1 overexpression affects numbers of BM- or spleen-conserved MP cells and whether megakaryopoietic development is impaired by excessive COMP-Ang1 depending on the amount generated. The *K14-Cre;c-Ang1* mice showed significantly fewer numbers of MP cells in the BM (Supplementary Fig. S5A, Fig. 2A) and spleen (Supplementary Fig. S5B, Fig. 2B) compared with littermate controls (*P* < 0.001). Numbers of circulating platelets (Fig. 2C), but not white blood cells (WBC; Fig. 2D), in the *K14-Cre;C-Ang1* mice were significantly less than that in their littermate controls (*P* < 0.001). The *Col2.3-Cre;c-Ang1* mice also exhibited significantly fewer numbers of BM MP cells (*P* < 0.05; Fig. 2E) and peripheral platelets (*P* < 0.001; Fig. 2G) without changes in the levels of spleen-conserved MP cells (Fig. 2F) and circulating WBC (Fig. 2H) compared with their littermate controls. Moreover, percentage of the Ki-67-positive MP cells in *Col2.3-Cre;c-Ang1* mice was comparable with that in their littermate controls (Fig. 2I, 2J). These results suggest that transgenic supplementation of COMP-Ang1 impairs megakaryopoietic development and platelet production, although the severity of the impairment is in part associated with the amount of COMP-Ang1. Our findings also indicate that the diminished level of circulating platelets in transgenic mice is not directly associated with a defective capacity of MP cells to proliferate.

### MP Cells Highly Express Tie2 more than Other HSPCs, and this is not Affected by Genetic Overexpression of COMP-Ang1

**Figure 3.**
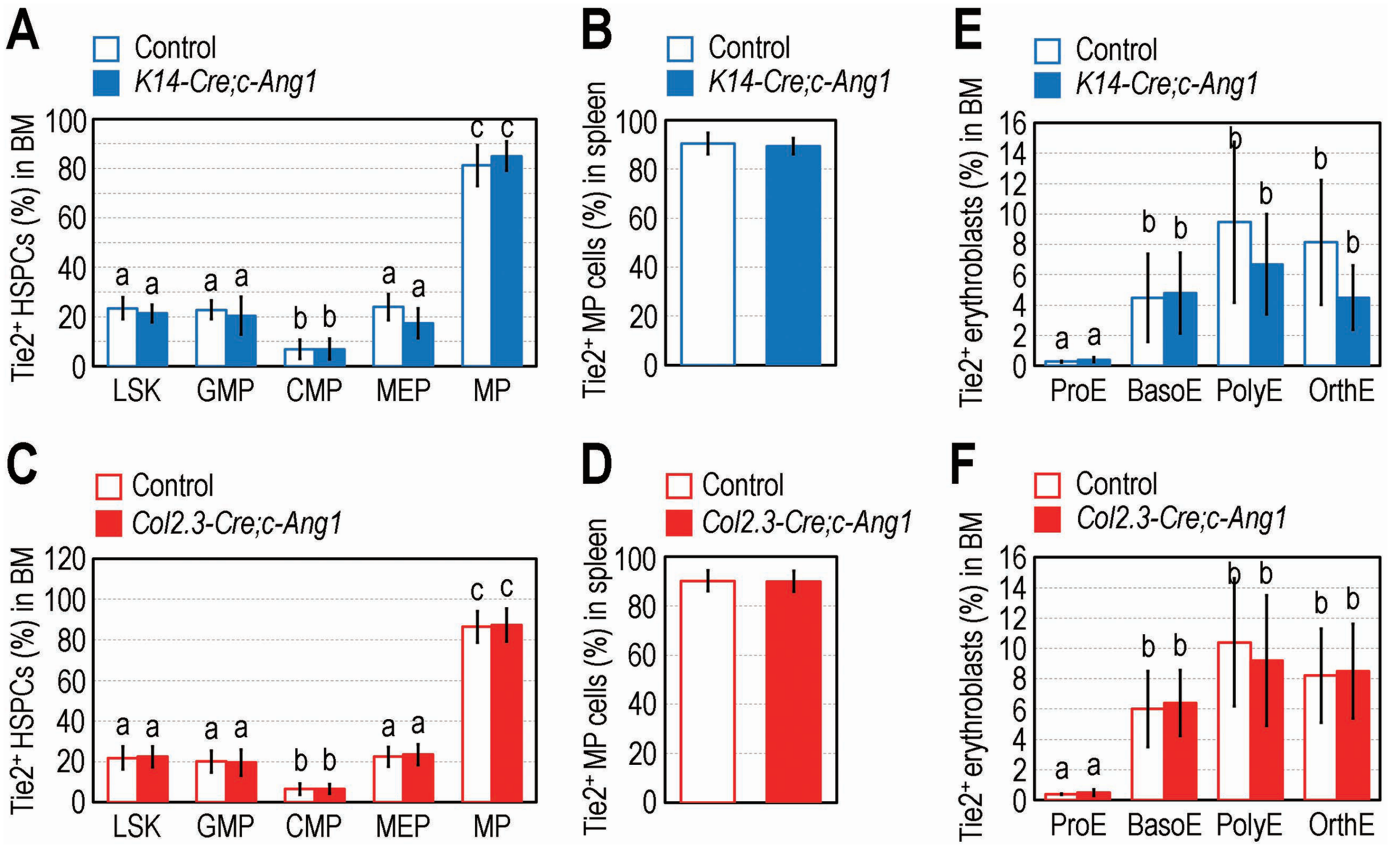
MP cells more highly express Tie2 than other HSPCs and erythroblasts. Mean percentages of Tie2-positive HSPCs in (**A**, **C**) BM and (**B**, **D**) spleen of (A, B) *K14-Cre;c-Ang1* and (C, D) *Col2.3-Cre;c-Ang1* mice and their littermate controls (*n* = 6). (**E)** BM erythroblasts (%) expressing Tie2 at four stages in *K14-Cre;c-Ang1* and (**F**) in *Col2.3-Cre;c-Ang1* mice along with their littermate controls (*n* = 8). Data represent mean ± S.D. Different superscripts^(a-c)^ indicate significant differences among the groups by one-way ANOVA.

We subsequently explored whether the impaired megakaryopoiesis in transgenic mice is related to the nature of HSPCs to express the Ang1-specific receptor, Tie2. The *K14-Cre;c-Ang1* mice showed similar levels of Tie2-expressing HSPCs in the BM to those in their littermate controls (Supplementary Fig. S6). In contrast, HSPCs exhibited different expression levels of Tie2 in relation to their cellular phenotypes. When the mean percentages of Tie2-positive HSPCs in the transgenic and control mice were calculated, we found that 6-23% of LSK, CMP, GMP, or MEP cells expressed the receptor, whereas more than 80% of MP cells showed Tie2 expression (Fig. 3A). Approximately 90% of spleen-conserved MP cells in the *K14-Cre;c-Ang1* mice and littermate controls expressed Tie2 (Fig. 3B). Similarly, the *Col2.3-Cre;c-Ang1* mice and their littermate controls expressed Tie2 in MP cells to higher levels than in LSK, CMP, GMP, and MEP cells in the BM (Fig. 3C) and spleen (Fig. 3D). Numbers of Tie2-positive BM erythroblasts in *K14-Cre;c-Ang1* and *Col2.3-Cre;c-Ang1* mice were also comparable with those in their littermate controls (Fig. 3E, 3F). However, Tie2 expression level in erythroblasts was apparently lower than that in BM- or spleen-conserved MP cells. These results indicate that among hematopoietic lineage cells, MP cells express high levels of Tie2, and thus megakaryopoietic development is sensitively affected more than erythropoiesis by supplemental COMP-Ang1, activation of the Ang1/Tie2 signaling axis, or both.

### Decreased Expression of GATA-1, But Not GATA-2, in MP Cells is Linked to Megakaryopoietic Impairment

**Figure 4.**
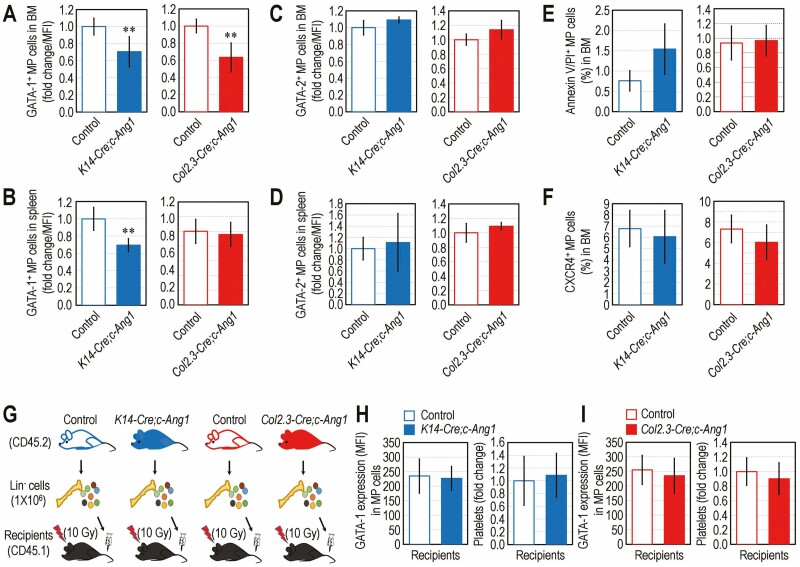
Transgenic overexpression of COMP-Ang1 inhibits the expression of GATA-1, but not GATA-2, in MP cells, and is not directly associated with HSC senescence. Flow cytometric results showing GATA-1-positive MP cells (MFI) in (**A**) BM and (**B**) spleen of transgenic mice and their littermate controls (*n* = 8). GATA-2-positive MP cells (MFI) in (**C**) BM and (**D**) spleen of transgenic mice and littermate controls (*n* = 5). BM-derived MP cells (%) positive for (**E**) Annexin V/PI or (**F**) CXCR4 in transgenic mice and littermate controls (*n* = 5). **G** BM-conserved lineage-negative cells (1 × 10^6^ cells) isolated from transgenic mice or their littermate controls (CD45.2) were transplanted into irradiated recipient mice (CD45.1). GATA-1 expression (MFI) in BM-conserved MP cells and level of circulating platelets in recipients transplanted with cells derived from (**H**) *K14-Cre;c-Ang1* or (**I**) *Col2.3-Cre;c-Ang1* mice were compared with those from their littermate controls at 5 months post-transplantation (*n* = 7). Data represent mean ± S.D. ^**^P < 0.01 vs. the controls by unpaired Student’s *t*-test.

As the GATA family proteins play essential roles in normal hematopoiesis,^24^ we explored whether the decreased frequency of MP cells in transgenic mice is correlated with a dysregulated expression of GATA-1 and GATA-2. Both *K14-Cre;c-Ang1* and *Col2.3-Cre;c-Ang1* mice exhibited a significant reduction (*P* < 0.01) in GATA-1 expression in BM-conserved MP cells compared with their littermate controls, respectively (Fig. 4A). The *K14-Cre;c-Ang1*, but not *Col2.3-Cre;c-Ang1* mice, showed a significant decrease (*P* < 0.01) in GATA-1 expression even in spleen-conserved MP cells compared with their littermate controls (Fig. 4B). However, transgenic mice exhibited GATA-2 expression in BM- and spleen-conserved MP cells similar to that in their littermate controls, respectively (Fig. 4C, 4D). The *K14-Cre;c-Ang1* and *Col2.3-Cre;c-Ang1* mice did not show any changes in the numbers of BM MP cells positive for Annexin V/PI (Fig. 4E) or anti-CXCR4 antibody (Fig. 4F) compared with their littermate controls. To explore whether HSC senescence is directly associated with loss of MP cells and circulating platelets, we performed transplantation assay by transplanting lineage-negative cells (10^6^ cells) isolated from transgenic mice and their littermate controls into conditioned recipient mice (Fig. 4G). Similar to the recipients transplanted with *K14-Cre;c-Ang1* mouse- or littermate control-derived cells (Fig. 4H), in recipients transplanted with the *Col2.3-Cre;c-Ang1* mouse-derived cells, GATA-1 expression in BM MP cells and circulating platelet level were comparable with those in the recipients with littermate control-derived cells (Fig. 4I). These findings indicate the association of megakaryopoietic defect with declined GATA-1 expression rather than GATA-2 expression, cell death, or dysregulation of the CXCR4-linked signaling in MP cells. These results also indicate that HSC senescence is not directly associated with the loss of GATA-1-expressing MP cells and circulating platelets.

### Ang1/Tie2 Signaling Axis is Linked to GATA-1 Expression in MP Cells and Platelet Production Impairment by Genetic Overexpression of COMP-Ang1

**Figure 5.**
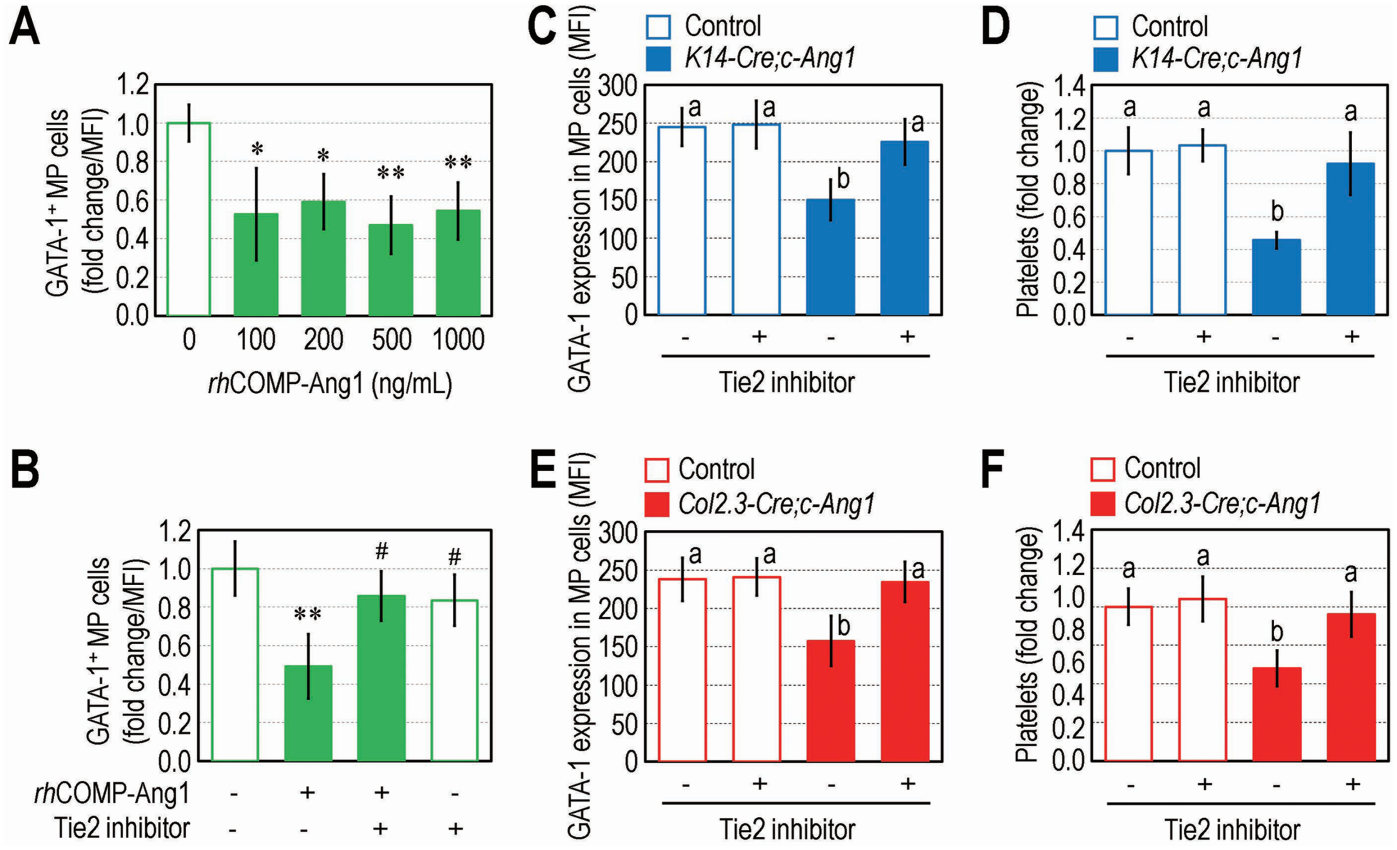
The Ang1/Tie2 signaling axis disturbs GATA-1 expression in MP cells and platelet production. (**A)** B6 mouse-derived MP cells were incubated with various concentrations (0-1000 ng/mL) of *rh*COMP-Ang1, and GATA-1-expressing MP cells (fold change of MFI) were flow cytometrically determined at 3 days post-incubation. (**B)** GATA-1 expression (MFI) in MP cells exposed to Tie2 kinase inhibitor (250 nM), 500 ng/mL *rh*COMP-Ang1, or both for 3 days. (**C**, **D**) *K14-Cre;c-Ang1*mice, (**E**, **F**) *Col2.3-Cre;c-Ang1* mice, and their littermate controls were injected peritoneally with PBS or Tie2 kinase inhibitor (10 mg/kg). (**C**, **E**) GATA-1-expressing MP cells (MFI) and (**D**, **F**) circulating platelets in the transgenic mice and littermate controls were determined (*n* = 8). Data represent mean ± S.D. ^*^P < 0.05; ^**^P < 0.01 vs. the untreated controls by unpaired Student’s *t*-test. ^#^*P* < 0.05 vs. the *rh*COMP-Ang1 treatment alone by unpaired Student’s *t*-test. Different superscripts^(a, b)^ indicate significant differences among the groups by one-way ANOVA.

As MP cells exhibit the highest expression of Tie2 among HSPCs and GATA-1 expression is linked to platelet production, we assessed whether the Ang1/Tie2 signaling axis is directly associated with GATA-1 expression and production of circulating platelets. To address this, we evaluated the in vitro impact of *rh*COMP-Ang1 on GATA-1 expression in MP cells. BM MP cells isolated from B6 mice were incubated in the presence of various concentrations (0–1,000 ng/mL) of *rh*COMP-Ang1, and GATA-1 expression in these cells was reduced in a dose-independent manner (Fig. 5A). The suppressed GATA-1 level (mean fluorescence intensity; MFI) in *rh*COMP-Ang1 (500 ng/mL)-exposed MP cells was almost completely recovered by treatment with the Tie2 kinase inhibitor (250 nM) (Fig. 5B). Tie2 inhibitor alone did not alter GATA-1 expression in the cultured MP cells. Based on the developmental cycle of megakaryocyte maturation and pro-platelet production in mice,^25^ we performed an in vivo inhibitory assay by peritoneally administering the Tie2 kinase inhibitor (10 mg/kg) into transgenic mice and their littermate controls. GATA-1 expression in BM MP cells of the *K14-Cre;c-Ang1* mice was recovered up to the level similar to that of untreated littermate control group (Fig. 5C). Circulating platelets in the *K14-Cre;c-Ang1* mice were also increased up to the level of littermate controls after injecting Tie2 kinase inhibitor (Fig. 5D). In addition, Tie2 inhibitor-administered *Col2.3-Cre;c-Ang1* mice exhibited GATA-1 expression (Fig. 5E) and circulating platelet levels (Fig. 5F) similar to those in untreated littermate controls. However, circulating RBC level was not affected by Tie2 inhibitor treatment in these transgenic mice and their littermate controls (data not shown). These results suggest that excessive presence of Ang1 or activation of the Ang1/Tie2 signaling axis impairs GATA-1 expression in MP cells and platelet formation, while the erythropoietic defect is not directly associated with the Ang1/Tie2 signaling axis.

### Consecutive Treatment with AMD3100 Causes Irrecoverable Senescence of BM HSCs in Mice

**Figure 6.**
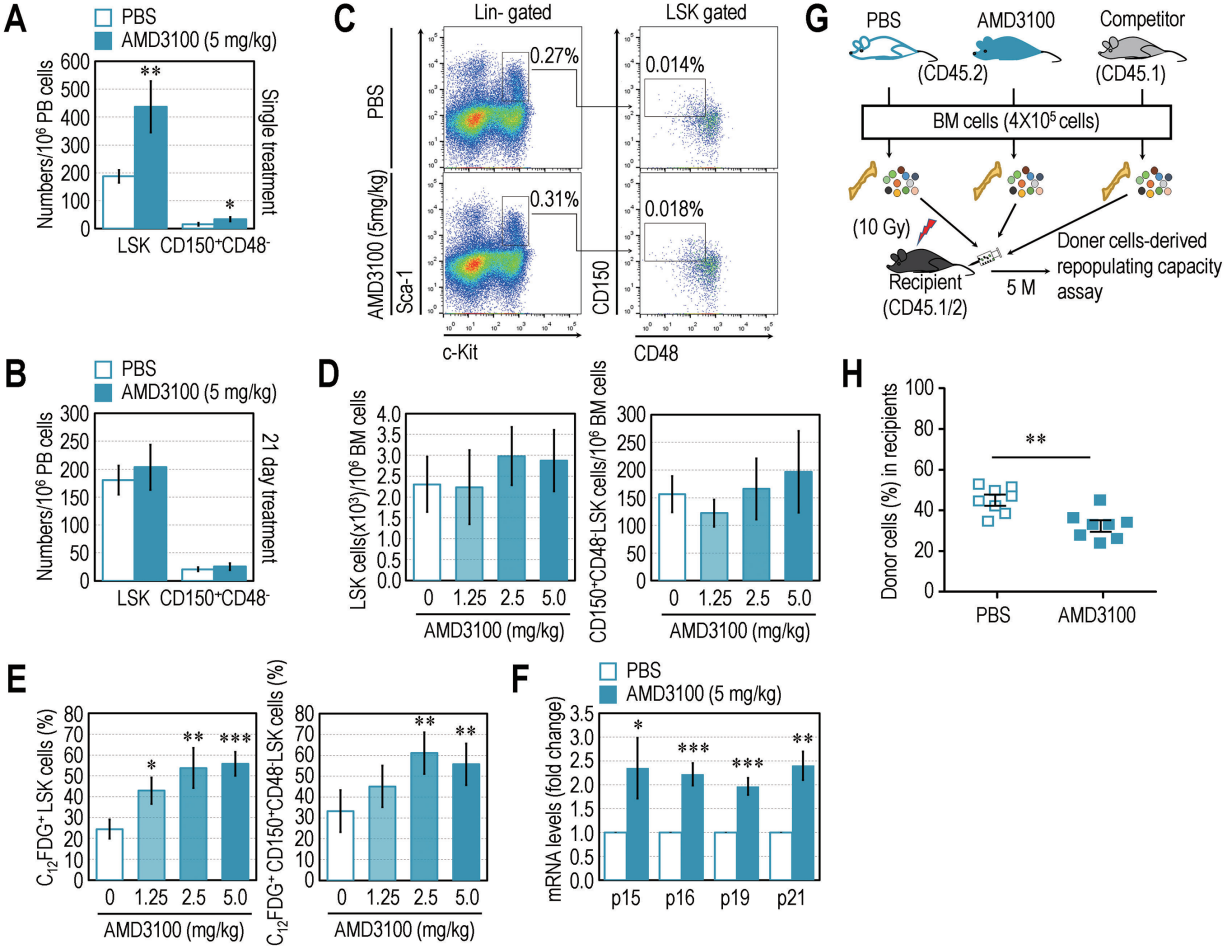
Disruption of the SDF-1/CXCR4 retention axis induces irrecoverable senescence of HSCs. B6 mice were administered with the indicated concentrations of AMD3100 or PBS for (**A**) 1 day or (**B**) 21 consecutive days, and numbers of LSK and CD150^+^CD48^-^LSK cells in PB were determined (*n* = 6). (**C**, **D)** BM frequencies of LSK and CD150^+^CD48^-^LSK cells in B6 mice after 12 h of the last administration (*n =* 6). B6 mice were also injected with the indicated concentrations of AMD3100 or PBS for 21 consecutive days. (**E)** Numbers of C_12_FDG-positive LSK and CD150^+^CD48^-^LSK cells (%) in BM (*n =* 6). (**F)** The mRNA levels of p15, p16, p19, and p21 in whole BM cells of PBS control and AMD3100 (5 mg/kg)-treated mice. **G** BM cells (4 × 10^5^ cells) from PBS control- or AMD3100 (5 mg/kg)-treated mice (CD45.2) were transplanted into conditioned recipient mice (CD45.1/2) together with BM cells (4 × 10^5^ cells) from competitor mice (CD45.1). (**H)** The donor cell-induced repopulating capacity in PB of the recipients was determined at 5 months post-transplantation (*n =* 8). Data represent mean ± S.D. ^*^P < 0.05; ^**^P < 0.01; ^***^P < 0.001 vs. the PBS controls by unpaired Student’s *t*-test (panels A, B, D, H) or by one-way ANOVA (panels D, E).

While single treatment of mice with AMD3100, a small and selective CXCR4 antagonist,^26^ rapidly increases numbers of circulating LSK and CD150^+^CD48^-^LSK cells, consecutive administration of AMD3100 leads to senescence of BM HSCs via disruption of the SDF-1/CXCR4 retention axis.^27^ Here, we explored the regulatory roles of the SDF-1/CXCR4 axis on BM retention and senescence of HSCs using B6 mice that were subcutaneously injected with AMD3100 (5 mg/kg) for 1 day or 21 consecutive days. The single injection, but not consecutive treatment, with AMD3100 facilitated mobilization of BM-conserved HSCs into PB in the mice (Fig. 6A, 6B). The long-term administration of AMD3100 did not change BM retention of LSK cells in B6 mice (Fig. 6C). Numbers of BM-conserved LSK and CD150^+^CD48^-^LSK cells were also not altered by consecutive AMD3100 treatment with different concentrations (0, 1.25, 2.5, and 5 mg/kg) (Fig. 6D). However, consecutive AMD3100 treatment increased numbers of BM LSK and CD150^+^CD48^−^LSK cells positive for C_12_FDG in a dose-dependent manner (Fig. 6E). B6 mice injected with 5 mg/kg of AMD3100 for 21 days exhibited significantly greater mRNA levels of p15 (*P* < 0.05), p16 (*P* < 0.001), p19 (*P* < 0.001), and p21 (*P* < 0.01) in BM cells compared with levels in PBS control (Fig. 6F). To verify whether HSC senescence induced by consecutive AMD3100 injection is recoverable, we determined the donor cell-derived repopulating capacity in lethally irradiated recipients after 5 months of transplantation (Fig. 6G). The recipients transplanted with BM cells derived from AMD3100-injected mice showed significantly (*P* < 0.01) fewer numbers of donor cells compared with the mice with PBS control-derived cells (Fig. 6H). These results indicate that similar to the phenotypes shown in *K14-Cre;c-Ang1* mice,^20^ consecutive AMD3100 injection induces irrecoverable senescence of BM HSCs in mice, and this is coupled with impaired retention of the SDF-1/CXCR4 axis.

### Long-Term Blockage of the SDF-1/CXCR4 Retention Axis Disrupts Erythrocyte Maturation and RBC Formation, but not Megakaryotic Development and Platelet Production, in Mice

**Figure 7.**
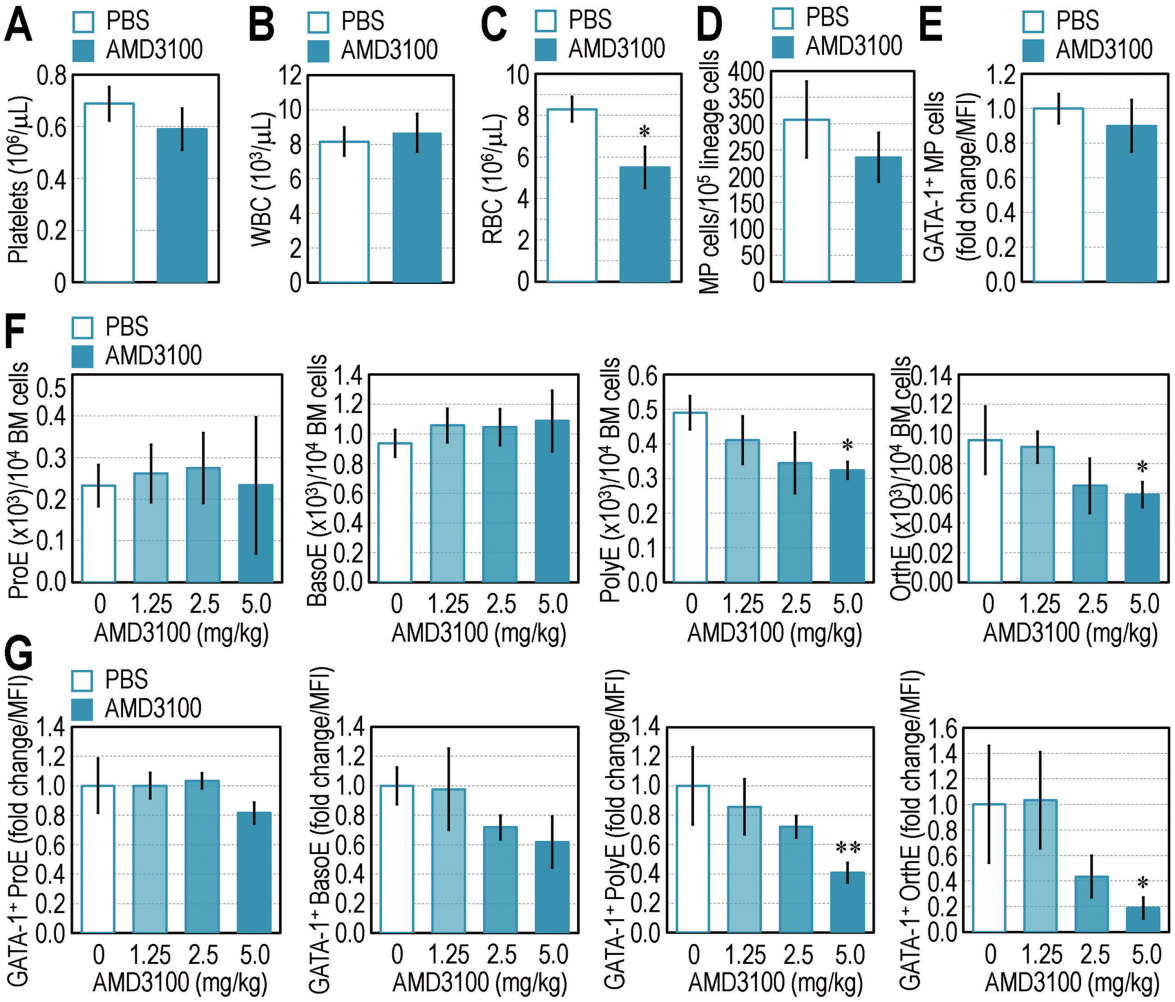
Long-term disruption of the SDF-1/CXCR4 axis diminishes numbers of erythroblasts, and this is correlated with GATA-1 expression. B6 mice were injected with PBS or 5 mg/kg of AMD3100 for 21 consecutive days. Levels of (**A**) circulating platelets, (**B**) WBC, and (**C**) RBC, as well as (**D**) number of BM MP cells and (**E**) their GATA-1 expression (fold change of MFI) were determined (*n* = 8). B6 mice were also administered with the indicated concentrations of AMD3100 for 21 consecutive days. (**F)** Numbers of BM-conserved erythroblasts at 4 stages and (**G**) GATA-1 expression (MFI) in these cells were analyzed (*n* = 8). Data represent mean ± S.D. ^*^P < 0.05; ^**^P < 0.01 vs. the PBS controls by unpaired Student’s *t*-test.

Based on the impacts of AMD3100 on BM retention and senescence of HSCs, we further examined the cellular mechanisms underlying how consecutive administration of AMD3100 affects megakaryopoietic and erythropoietic development in B6 mice. Consecutive injection of AMD3100 (5 mg/kg) did not alter circulating numbers of platelets (Fig. 7A) and WBC (Fig. 7B), but significantly (*P* < 0.05) reduced peripheral RBC level in the mice (Fig. 7C). B6 mice administered with 5 mg/kg AMD3100 for 21 days did not show any differences in number of BM-conserved MP cells (Fig. 7D) and GATA-1 expression (Fig. 7E) in these MP cells compared with those with PBS-received controls. To better understand the reason by which AMD3100 treatment impairs RBC production in B6 mice, we administered mice with different concentrations (0-5 mg/kg) of AMD3100 or PBS for 21 days and evaluated the numbers of BM erythroblasts and GATA-1 expression in BM erythroblasts by phenotypically defining the cells at 4 stages. BM numbers of PolyE and OrthE, but not ProE and BasoE, were significantly (*P* < 0.05) reduced in B6 mice injected consecutively with 5 mg/kg AMD3100 compared with numbers in the PBS control (Fig. 7F). A decrease in the number of BM erythroblasts in AMD3100-administered mice was tightly associated with the proportion of GATA-1-expressing cells (Fig. 7G). These results indicate that disruption of the SDF-1/CXCR4 retention axis causes abnormal BM retention and senescence of HSCs and damages erythropoietic maturation and RBC production, rather than platelet production. Our results also indicate a correlation between numbers of erythroblasts and their GATA-1 expression specifically at the late stage of erythrocytic maturation.

## Discussion

The SDF-1/CXCR4 axis plays important roles in maintaining the HSC pool and in regulating HSC mobilization and homing.^28-30^ Single AMD treatment rapidly promotes HSPC mobilization into PB, but its long-term administration may induce hematopoietic damage via impaired retention of the SDF-1/CXCR4 axis.^31-33^ Here, we demonstrate that different from *K14-Cre;c-Ang1* mice, *Col2.3-Cre;c-Ang1* mice did not exhibit impaired BM retention and senescence of HSCs and anemic disorder, and these differences were correlated with the SDF-1/CXCR4 retention axis. Our results also indicate that disruption of the SDF-1/CXCR4 retention axis mediates HSC senescence and erythropoietic impairment, whereas it does not directly disturb megakaryopoiesis and platelet production. Although the CXCR4 antagonist AMD3100 had no a direct relationship with the transgenic Ang1 overexpression models, our data may support crucial roles of SDF-1/CXCR4 axis on hematopoietic development.

Activation of the Ang1/Tie2 signaling axis is required for megakaryocyte maturation and platelet formation.^8^ Our experiments in transgenic mice indicate that genetic overexpression of COMP-Ang1 impairs megakaryocytic development and platelet production regardless of the promoter. In vitro treatment of rhCOMP-Ang1 in BM cells including Tie2-positive MP cells decreased GATA1-positive MP cells (%). Although this decrease was not correlated with the concentration of rhCOMP-Ang1, several experiments using Tie2 inhibitor indicated important role of Ang1/Tie2 signaling axis on the regulation of GATA1 expression in MP cells. Consequently, our results support that megakaryotic defects in the transgenic mice are closely associated with the Ang1/Tie2 signaling axis, as well as the nature of MP cells to highly express Tie2. In addition, in vitro and in vivo experiments using *rh*COMP-Ang1, the Tie2 inhibitor, or both suggest that activation of the Ang1/Tie2 signaling axis results in defects in circulating platelet formation, rather than RBC production.

Transcription factors GATA-1 and GATA-2 play central roles in the development and maturation of megakaryocytes and erythrocytes into platelets and RBC.^20,34-37^ In addition to the impacts of GATA-1 on erythrocytic maturation and RBC production,^38-40^ considerable findings support that the lack of GATA-2 causes a defective hematopoiesis, whereas its overexpression stimulates megakaryocyte differentiation and impairs erythroid differentiation.^41^ To further understand the cellular mechanisms by which transgenic overexpression of COMP-Ang1 induces hematopoietic defects, specifically thrombocytopenic and anemic disorders, we determined the numbers and expression of transcription factors GATA-1, GATA-2, or both in MP cells and erythroblasts in transgenic mice, irradiated recipients, or AMD3100-injected B6 mice. Our results suggest that numbers of MP cells and erythroblasts are closely associated with their expression of GATA-1, but not GATA-2. Our data indicate an association of GATA-1 with excessive Ang1-mediated impairments in platelets and RBC production. As shown by the unchanged numbers of MP cells positive for Ki-67 or Annexin V/PI, our findings also indicate that impaired platelet formation in transgenic mice is not directly associated with an alteration of MP cell death and proliferation.

While GATA-1 expression appeared to be tightly correlated with the levels of MP cells and erythroblasts in transgenic mice, our results also strongly suggest the existence of different mechanisms by which GATA-1 expression in these cells is affected in relation to the amount of Ang1. One report showed that megakaryopoiesis is more sensitively affected by dysregulated GATA-1 function than erythropoiesis.^42^ Based on the effects of Tie2 inhibitor on GATA-1 expression and production of platelets in transgenic mice, we postulated that activation of the Ang1/Tie2 signaling axis may directly cause thrombocytopenic damage rather than anemia via dysregulated GATA-1 expression in MP cells. In contrast, our results indicate that anemic disorder induced in transgenic mice is related to impaired retention of the SDF-1/CXCR4 axis along with decreased GATA-1 expression in erythroblasts at late stages. The current findings along with previous reports^19,20^ also suggest that the impaired SDF-1/CXCR4 retention axis in transgenic mice is correlated with the amount of COMP-Ang1 generated. It is important to consider that HSC senescence is not directly associated with megakaryocyte maturation and platelet formation, but it rather mediates erythropoietic disorder.^43^ In addition, inflammatory responses repress GATA-1 expression and erythropoietic development.^36^ Taken together, the current findings with previous reports^19,20^ indicate that in addition to the dysregulated expression of SDF-1 and GATA-1, the increases in inflammatory cytokines, BM niche injury, and HSC senescence are orchestrated with defected erythrocyte maturation and RBC production under systemic overexpression of COMP-Ang1.

In summary, numerous studies have shown therapeutic potential of COMP-Ang1, an engineered angiopoietin-1 variant, at both vascular and systemic levels.^44^ Administration of rhCOMP-Ang1 via a local delivery or adenoviral transfection system exhibits beneficial effects on vasculogenesis, angiogenesis, and regenerative medicines. It has been also considered that a prolonged and persisted supplementation with COMP-Ang1 exert further great potency in its use for medicinal and pharmacological application. We could not exclude the fact that the transgenic mice used in this study are artificial models and thus can evoke a limitation in a clinical application of the current findings. However, our results provide evidence that an appropriate activation of the Ang1/Tie2 signaling and/or a suitable level of Ang1 via a local supplementation of rhCOMP-Ang1, but not its genetic overexpression, are required for the maintenance of HSC functions as well as for production of megakaryocytes and erythroid progenitor-derived cells.

## Conclusion

This study demonstrates that long-term supplementation with COMP-Ang1 by its transgenic overexpression induced impaired BM retention and senescence of HSCs and led to defects in erythropoiesis, megakaryopoiesis, or both depending on its systemic and local expression (Supplementary Fig. S7). The main mechanisms by which transgenic overexpression of COMP-Ang1 mediates anemic and thrombocytopenic disorders involve pivotal roles of the Ang1/Tie2 signaling and SDF-1/CXCR4 retention axis. Our findings also support a correlation between GATA-1 expression and the numbers of MP cells and erythroblasts.

## Supplementary Material

sxac080_suppl_Supplementary_Material

## Data Availability

The data that support the findings of this study are available on request from the corresponding author.
