# Supplementary material for: Local and Systemic Overexpression of COMP-Ang1 Induces Ang1/Tie2-Related Thrombocytopenia and SDF-1/CXCR4-Dependent Anemia
Source: Stem Cells. 2022 Nov 11;41(1):93–104. doi: 10.1093/stmcls/sxac080 (PMC9887089; doi:10.1093/stmcls/sxac080)
Supplement: sxac080_suppl_Supplementary_Material [file sxac080_suppl_supplementary_material.pdf]

**Online Supporting Information**

**Supplemental methods**

## 2.1 Measurement of blood vessel formation

Phenotypes of blood vessel formation in transgenic mice and littermate controls were evaluated according to the methods previously described.[20] In brief, the aorta of anesthetized mice was adjusted to systemic perfusion with 10 mL of 0.9% NaCl and 200 U/mL heparin at 37°C and then to manual perfusion with yellow Microfil® (MV-122) at a rate of 3 mL/min. After ligation of left ventricle and right atrium and excision of knee joints, the femurs were treated with Decalcifying Solution-Lite (Sigma-Aldrich Co. LLC). Finally, tissue samples were scanned with a Skyscan 1076 (Skyscan, Bruker, Belgium) at a pixel size of 9 μm, and images were reconstructed using CtVox software.

## 2.2 Transplantation assay using transgenic mice and their littermate controls

Lineage-negative cells ($1 \times 10^6$ cells) were isolated from the BM of transgenic mice and their littermate controls (CD45.2) using auto-MACS instrument (Miltenyi Biotec) and lineage cell depletion kit (cat.#130-090-858; Miltenyi Biotec). These cells were transplanted into conditioned recipient mice (CD45.1) that were lethally irradiated with 10 Gy by regulating dosage time that was based on the radioactive half-life of γ-rays on a rotating platform (Model 109-85 series-JL Shepherd & associates, San Fernando). GATA-1 expression in MP cells and levels of circulating

platelets and RBC in recipients were determined by flow cytometry at 5 month post-transplantation that is a stable period that transplanted cells are being repopulated and differentiated in the recipients. In all experiments to assay the expression of GATA-1 and/or GATA-2, results were represented as mean fluorescence intensity (MFI) or fold change of MFI of the transcription factor-positive cells.

## 2.3 Annexin V and propidium iodide (PI) staining

After removing RBC, BM-conserved cells of transgenic mice and their littermate controls were incubated with antibodies specific to MP cells followed by treatment with aqueous buffered solution of FITC-labeled Annexin V (200 ng/mL) and propidium iodide (PI, 300 ng/mL) at room temperature for 20 min. Frequencies of cells were analyzed using flow cytometer, and the scatter signals of Annexin V- and PI-positive cells were evaluated after sequentially gating cell populations using FlowJo software program.

## 2.4 Blood test

PB samples were isolated from transgenic mice, littermate controls, or B6 mice and collected into Vacutainer plastic tubes coated with $K_2EDTA$. Automated complete blood cell counter (Sysmex XE-2100; TOA Medical Electronics Co., Kobe, Japan) was used to measure the levels of RBC (number/μL), white blood cells (WBC; number/μL), and platelets (number/μL).

**2.5 Immunohistochemistry for CD31 expression**

Immunohistochemistry was performed using the Histostain Plus Rabbit Primary kit (Zymed Laboratories) according to the manufacturer's instruction. Briefly, hind limbs were dissected from transgenic mice and their littermate controls. After fixation and decalcification, the tissue samples were sectioned at a thickness of 5 μm followed by subsequent procedures for xylene treatment and descending serial hydration. Finally, slides were incubated with mouse-anti-CD31 antibody (cat.#ab9498; Abcam) followed by observation using a light microscope (Carl Zeiss, Ostalbkreis, Germany).

**2.6 Enzyme-linked immunosorbent assay (ELISA)**

SDF-1 level in BM supernatants of transgenic mice and their littermate controls was evaluated by ELISA using SDF-1-specifc mouse ELISA kit (Abcam). Levels of interleukin (IL)-1α, IL-6, interferon (IFN)-γ, and tumor necrosis factor (TNF)-α in BM supernatants of transgenic mice and their littermate controls were also measured using Multi-Analyte ELISArray Kits (QIAGEN Sciences). All procedures followed the manufacturer's instructions.

**Supplementary figures**

Figure S1

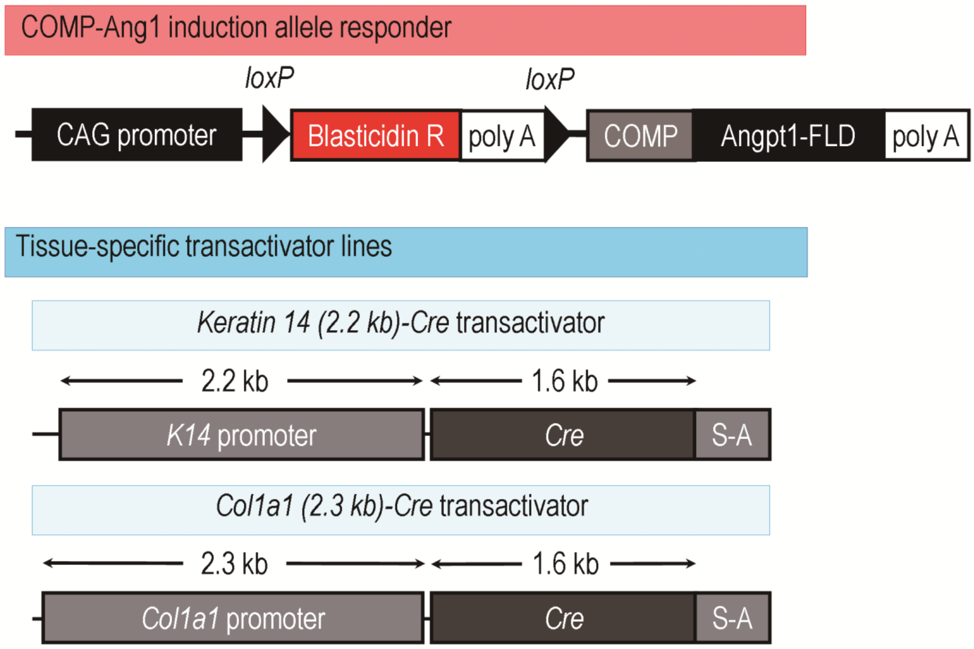

Figure S2

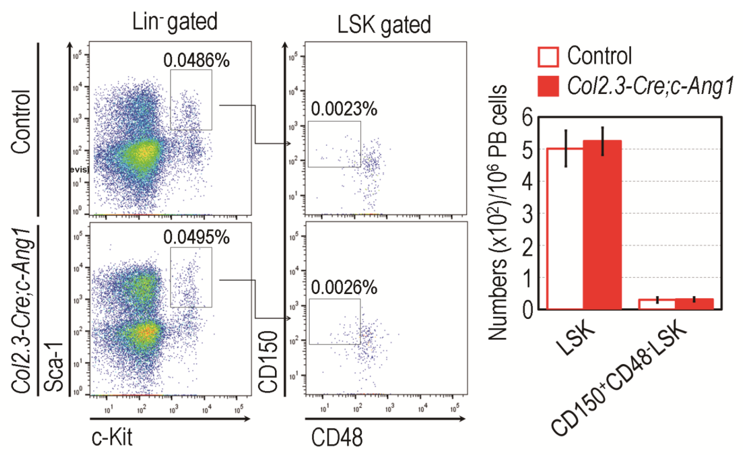

Lin⁻ gated
LSK gated
Control
Col2.3-Cre;c-Ang1
Sca-1
c-Kit
CD150
CD48
0.0486%
0.0495%
0.0023%
0.0026%
Control
Col2.3-Cre;c-Ang1
Numbers (x10²)/10⁶ PB cells
0
1
2
3
4
5
6
LSK
CD150⁺CD48⁻LSK

Figure S3

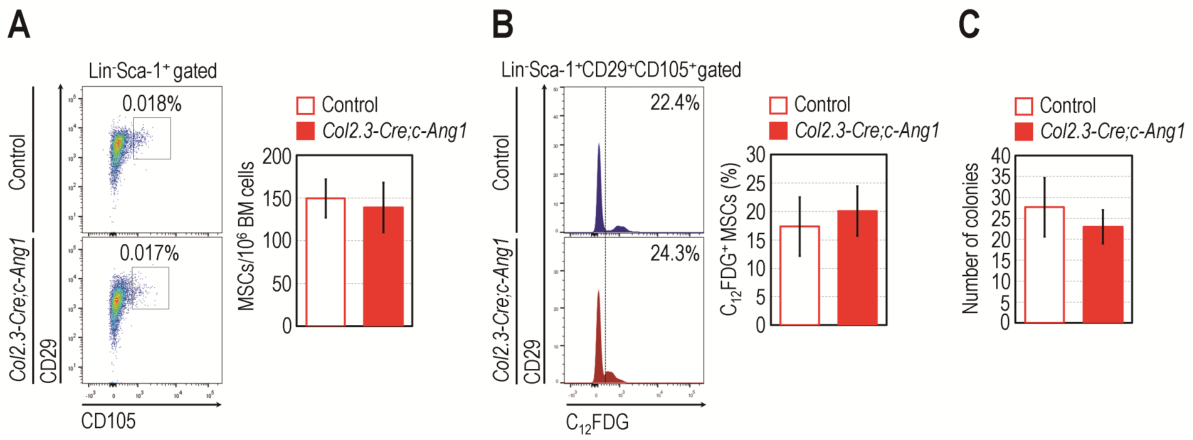
A
Lin-Sca-1+ gated
Control
0.018%
Col2.3-Cre;c-Ang1
0.017%
CD29
CD105
Control
Col2.3-Cre;c-Ang1
MSCs/10^6 BM cells
200
150
100
50
0
B
Lin-Sca-1+CD29+CD105+gated
Control
22.4%
Col2.3-Cre;c-Ang1
24.3%
CD29
C12FDG
Control
Col2.3-Cre;c-Ang1
C12FDG+ MSCs (%)
30
25
20
15
10
5
0
C
Control
Col2.3-Cre;c-Ang1
Number of colonies
40
35
30
25
20
15
10
5
0

Figure S4

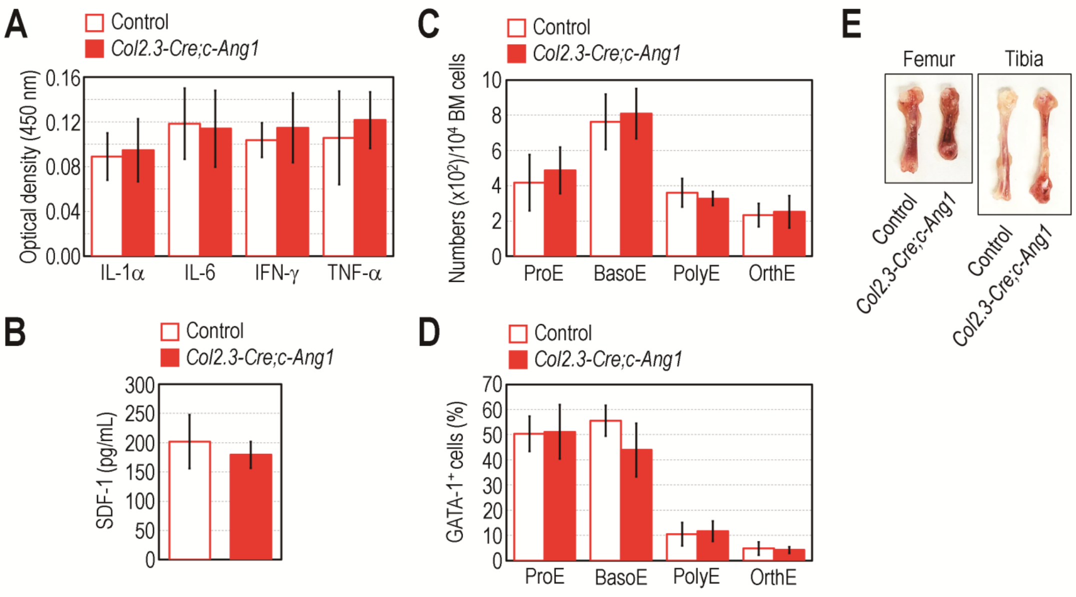

A
Control
Col2.3-Cre;c-Ang1
Optical density (450 nm)
0.16
0.12
0.08
0.04
0.00
IL-1α
IL-6
IFN-γ
TNF-α
B
Control
Col2.3-Cre;c-Ang1
SDF-1 (pg/mL)
300
250
200
150
100
50
0
C
Control
Col2.3-Cre;c-Ang1
Numbers (x10²)/10⁴ BM cells
10
8
6
4
2
0
ProE
BasoE
PolyE
OrthE
D
Control
Col2.3-Cre;c-Ang1
GATA-1⁺ cells (%)
70
60
50
40
30
20
10
0
ProE
BasoE
PolyE
OrthE
E
Femur
Tibia
Control
Col2.3-Cre;c-Ang1
Control
Col2.3-Cre;c-Ang1

Figure S5

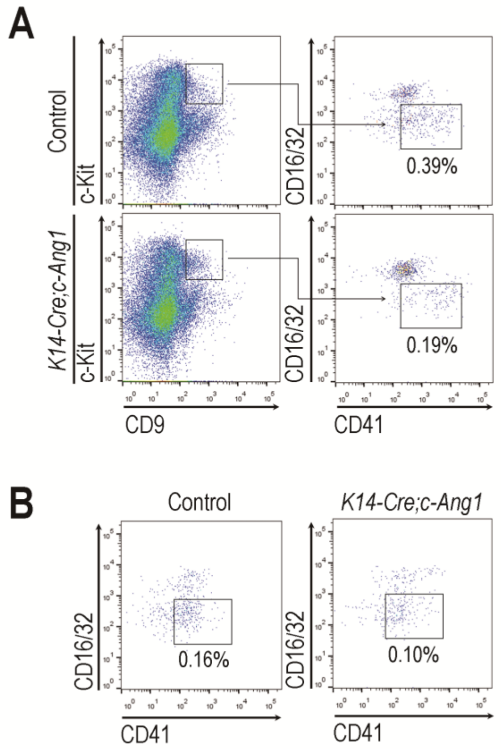
A
Control
c-Kit
CD16/32
0.39%
K14-Cre;c-Ang1
c-Kit
CD16/32
0.19%
CD9
CD41
B
Control
K14-Cre;c-Ang1
CD16/32
0.16%
CD16/32
0.10%
CD41
CD41

Figure S6

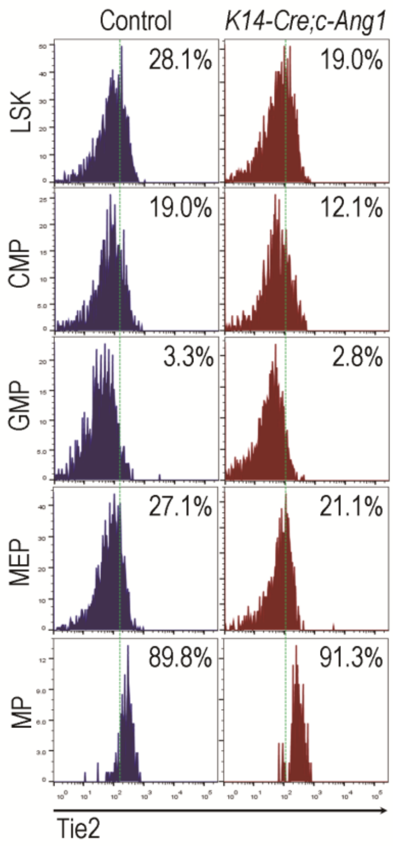
Control
K14-Cre;c-Ang1
LSK
CMP
GMP
MEP
MP
28.1%
19.0%
19.0%
12.1%
3.3%
2.8%
27.1%
21.1%
89.8%
91.3%
Tie2

Figure S7

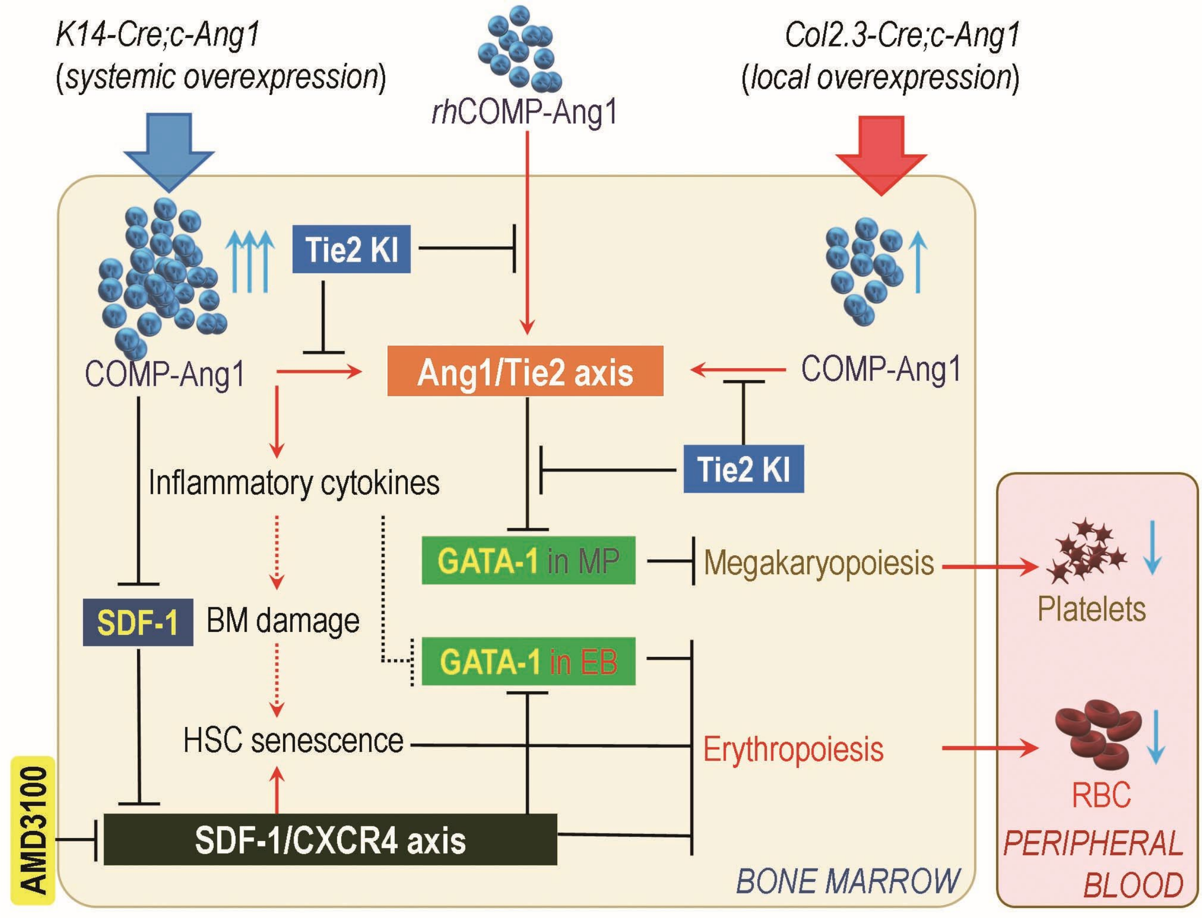
K14-Cre;c-Ang1
(systemic overexpression)
rhCOMP-Ang1
Col2.3-Cre;c-Ang1
(local overexpression)
Tie2 KI
COMP-Ang1
Ang1/Tie2 axis
COMP-Ang1
Inflammatory cytokines
Tie2 KI
GATA-1 in MP
Megakaryopoiesis
Platelets
SDF-1
BM damage
GATA-1 in EB
HSC senescence
Erythropoiesis
RBC
AMD3100
SDF-1/CXCR4 axis
BONE MARROW
PERIPHERAL BLOOD

## Supplemental figure legends

**Figure S1. Strategy of tissue-specific COMP-Ang1 inducible transgenic mice generation.**

**Figure S2. *Col2.3-Cre;c-Ang1* mice do not exhibit any changes in numbers of peripheral LSK and $CD150^{+}CD48^{-}$LSK cells.**

Numbers of spleen-conserved LSK and $CD150^{+}CD48^{-}$LSK cells in the transgenic mice and littermate controls ($n = 8$). *P*-values were calculated by unpaired Student's *t*-test. Data represent mean ± S.D.

**Figure S3. The *Col2.3-Cre;c-Ang1* mice do not show any alterations in number and function of BM MSCs.**

**A** Numbers of BM-conserved MSCs or **B** $C_{12}$FDG-positive MSCs in *Col2.3-Cre;c-Ang1* mice and littermate controls at 3 weeks of age ($n = 8$). **C** MSCs ($3 \times 10^4$ cells per dish) were isolated from BM of the *Col2.3-Cre;c-Ang1* mice and littermate controls at 3 weeks of age, and after 12 days of incubation in 35 mm dishes, number of MSC-derived colonies containing more than 50 cells per colony was counted after staining with 0.5% crystal violet dissolved in 100% methanol. *P*-values were calculated by unpaired Student's *t*-test. Data represent mean ± S.D.

**Figure S4. Local overexpression of COMP-Ang1 does not alter inflammatory cytokine production, SDF-1 induction, and erythropoietic development.**

**A** Levels of IL-1α, IL-6, IFN-γ, and TNF-α and (**B**) SDF-1 in BM supernatants of the *Col2.3-Cre;c-Ang1* mice and littermate controls ($n$ = 6). **C** Numbers of and **D** GATA-1 expression in BM erythroblasts at four stages in the transgenic mice and littermate controls ($n$ = 8). **E** Photographs showing the femur and tibia isolated from the transgenic mice and littermate controls. $P$-values were calculated by unpaired Student's $t$-test. Data represent mean ± S.D.

**Figure S5. Flow cytometric analysis showing numbers of MP cells in *K14-Cre;c-Ang1* mice and littermate controls.**

Numbers of MP cells conserved in (**A**) BM and (**B**) spleen of *K14-Cre;c-Ang1* mice and their littermate controls were analyzed by flow cytometry. Representative results from more than 8 different samples are shown.

**Figure S6. Flow cytometric results showing Tie2-positive HSPCs in *K14-Cre;c-Ang1* mice and their littermate controls.**

Expression level of Tie2 in BM-conserved HSPCs of the *K14-Cre;c-Ang1* mice and littermate controls was analyzed by flow cytometry. Representative results from 6 different samples are shown.

**Figure S7. A schematic illustration indicating the possible mechanisms by which transgenic systemic and local overexpression of COMP-Ang1 disturbs megakaryopoietic and erythropoietic developments in relation to its amounts generated.**
